# Impact of *Mycobacterium tuberculosis* complex lineages as a determinant of disease phenotypes from an immigrant rich moderate tuberculosis burden country

**DOI:** 10.1186/s12931-018-0966-x

**Published:** 2018-12-27

**Authors:** Bright Varghese, Mushira Enani, Abdulrahman Alrajhi, Sameera Al Johani, Ali Albarak, Sahar Althawadi, Noura Elkhizzi, Hawra AlGhafli, Mohammed Shoukri, Sahal Al - Hajoj

**Affiliations:** 1 Department of Infection and Immunity, MBC-03, King Faisal Specialist Hospital and Research Centre, Post Box # 3354, Riyadh, 11211 Saudi Arabia; 2 Medical Specialties Department, King Fahad Medical City, Riyadh, Saudi Arabia; 3 Department of Medicine, King Faisal Specialist Hospital and Research Centre, Riyadh, Saudi Arabia; 4 Department of Microbiology, King Abdul Aziz Medical City, Riyadh, Saudi Arabia; 5 Department of Medicine, Prince Sultan Military Medical City, Riyadh, Saudi Arabia; 6 Department of Pathology and Laboratory Medicine, King Faisal Specialist Hospital and Research Centre, Riyadh, Saudi Arabia; 7 Department of Microbiology, Prince Sultan Military Medical City, Riyadh, Saudi Arabia; 8 National Biotechnology Centre, King Faisal Specialist Hospital and Research Centre, Riyadh, Saudi Arabia

**Keywords:** Mycobacterium tuberculosis complex, Saudi Arabia, Disease phenotypes, Tuberculosis, Extrapulmonary tuberculosis

## Abstract

**Background:**

Growing evidences suggested that the *Mycobacterium tuberculosis* complex (MTBC) lineages can determine the clinical outcome of pulmonary and extra-pulmonary tuberculosis. However, limited data only available revealing such association of bacterial genotypes and clinical phenotypes from immigrant rich countries.

**Methods:**

A multicenter study has been carried out on a collection of 2092 (1003 extrapulmonary and 1089 pulmonary) MTBC isolates. Genotyping of all the isolates were carried out by spoligotyping and 24 loci based MIRU-VNTR typing.

**Results:**

Demographically domination of young Saudi nationals (61.4%) and men (61.2%) were found in this cohort. Lymph nodes (62.4%) and gastrointestinal sites (16.7%) were the most common anatomical sites of infection. The predominant lineages were Delhi/CAS (26.9%), EAI (14.2%) and Ghana (9.9%). *Mycobacterium africanum* type I and II were reported for the first time in the country among extrapulmonary cases. ‘Ancestral’ lineages *M.bovis* (OR-5.22; 95% CI-2.23-8.22, *p*- < 0.001) and Delhi/CAS (OR-0.57; 95% CI-0.411-0.734, *p*- < 0.001) were directly associated with lymph node tuberculosis and gastrointestinal tuberculosis (*M. bovis*-OR-0.33; 95% CI-0.085-0.567, p-0.001 and Delhi/CAS-OR-1.87; 95% CI-1.22-2.53, *p*- < 0.001) respectively. Among the ‘Modern’ lineages, EAI showed significant association to central nervous system tuberculosis (OR-1.98; 95% CI-0.76-3.19, p-0.04) and Uganda-I to gastrointestinal tuberculosis (OR-2.41; 95% CI-0.77-4.06, p-0.02).

**Conclusions:**

The findings substantially contribute to the emerging evidences that MTBC lineages influence disease phenotypes and epidemiological consequences.

**Electronic supplementary material:**

The online version of this article (10.1186/s12931-018-0966-x) contains supplementary material, which is available to authorized users.

## Background

Tuberculosis (TB) caused by *Mycobacterium tuberculosis complex* (MTBC) remains as a major public health challenge despite of implementing several control programs [1]. World Health Organization (WHO) estimated in 2016 that, there were 10.6 million newly diagnosed TB cases including 490,000 multidrug resistant cases and 1.4 million deaths [1]. The MTBC primarily infects the lungs, but virtually can affect any site in the body. Extrapulmonary TB (EPTB) is a difficult form of TB to diagnose and treat and results in high mortality and morbidity. Recent national level report from Saudi Arabia, showed 25.6% of annually reported new TB cases were EPTB [2]. Interestingly, the annual EPTB incidence rate in Saudi Arabia is comparatively higher than developed countries, where EPTB incidences are even increasing steadily [3, 4].

In EPTB, lymph nodes, meninges, kidney, spine and joints/bones are mostly affected. However, TB in pericardium, peritoneum, pleura, liver, intestine, skin, genitourinary tracts, spleen, ileum, cecum, eyes, breast, penis, are also occasionally reported [5]. EPTB is mostly observed as a key indicator of immunosuppression. To date, bacterial determinants of pulmonary TB (PTB) or EPTB have not been clearly estimated, although large attention is directed to explore the host and pathogen determinants. There have been controversies in correlating specific lineages with morbidity and mortality due to TB infection. Several previous studies revealed conflicting evidences on the association of MTBC phylogenetic lineages and site of infection [6, 7]. However, such analysis was scarce from the Middle Eastern countries including Saudi Arabia. Saudi Arabia has a highly diverse population structure including 10.4 million immigrant workers from around the world. In addition, annually the country receives 10 million pilgrims to the Islamic holy cities. This population diversity was reflected highly on the spectrum of MTBC lineages in the country, with the presence of almost all defined lineages including indigenous strains [8–10]. Although, a recent study reported the lineage spectrum of MTBC in PTB and EPTB cases in the country, no detailed systematic analysis was carried out to date [8]. Therefore, for the first time in the country a multicenter study on a large cohort of pulmonary and extrapulmonary TB cases has been carried out to analyze the impact of MTBC lineages towards disease phenotypes.

## Methods

### Study design

The study has been carried out in four major referral hospitals in Riyadh (Central Province). During August 2014–July 2016, all cases diagnosed with active TB and culture positivity were included. A collection of 1003 non-repetitive extrapulmonary MTBC *(M.tuberculosis, M.bovis, M.africanum*) culture isolates were successfully enrolled. In addition, 1089 pulmonary MTBC isolates from sputum, gastric aspirates, bronchioalveolar lavage and lung tissues were included as a control. Standard data collection form with information on age, gender, nationality, infection site, AFB smear results and HIV status were filled during the isolate collection from each study centers. All culture negative cases were excluded from enrollment. Disseminated TB and miliary TB cases were also excluded, while pleural TB cases were included as PTB. The study has been reviewed and approved by the Office of Research Affairs at King Faisal Specialist Hospital and Research Centre, Riyadh.

### Sample collection, processing, and identification

*Mycobacterium tuberculosis* complex isolates from extrapulmonary sites were collected from EPTB diagnosed cases in the participating four study sites. Pulmonary isolates were selected from the culture collection of the research facility. Genomic DNA was extracted from all the isolates by using PrepIT MAX kit (DNA Genotek, Ottawa, Canada) according to the manufacturer’s instructions. Primary genotyping of all isolates were carried out by using the commercially available spoligotyping assay (Ocimum Biosolutions, Hyderabad, India). The 24 loci based MIRU-VNTR typing based on quadruplex PCR (Genoscreen, Lille, France) was carried out according to the manufacturer’s instructions in a 3730xl DNA Analyzer (Life Technologies, CA, USA).

### Data analysis

#### Definition of clinical phenotypes

Two major grouping of isolates were followed based on the site of infection, pulmonary and extrapulmonary. Based on the proportion of cases, extrapulmonary group were further classified into six groups namely lymph nodes, gastrointestinal (gastrointestinal tract, solid visceral organs), central nervous systems (CNS), bone and joints, skin, and urogenital. All other sites with lower proportions were grouped into “others”.

#### Genotyping data

The spoligotyping membranes were scanned and data were converted into numerical octal codes. The alleles of MIRU-VNTR types were primarily identified by using the Genemapper version-4.0 (Applied Biosystems, CA, USA). The spoligo-octal signatures and the MIRU-VNTR allele profiles were combined in Microsoft Excel and submitted to the international online MIRU_VNTR database (www.miru-vntrplus.org) for a combined best-match and phylogenetic tree-based analysis. We followed the phylogenetic classification based on large sequence polymorphisms to make combined discussion of study findings. The ‘Ancestral’ lineages included Delhi/CAS, *M. bovis* and *M. africanum*, while ‘Modern’ lineages included EAI, Beijing and Euro-American respectively [11].

The statistical analysis of data was carried out by using the SPSS version-20 package (IBM, NY, USA). Major strain lineages and different sites of infections were compared by Chi square test and regression analysis, estimating the odds ratio and 95% confidence interval. The *p* value ≤0.05 was considered as statistically significant.

## Results

During the study period, 1003 extrapulmonary isolates and 1089 pulmonary isolates were enrolled. Matching the age group of patients against the PTB and EPTB incidence showed significant variations with statistical associations. The age group below 14 years showed a statistically significant predominance of EPTB (*P* value < 0.0001).On the other hand, PTB incidence was more significant among the age group 15–29 and 49–59. Overall, 76.6% of EPTB cases were found with an age below 45 years. Among, EPTB patients 83.3% were Saudi nationals. Demographics and other patient characteristics have been summarized in Table 1 and Additional file 1. Distribution major lineages among the study population based on their nationality is depicted in Additional file 2.

**Table 1:** Demographic and clinical summary of study subjects

Parameters	Extrapulmonary N (%)	Pulmonary N (%)	*P* value
	*N* = 1003	*N* = 1089	
Age group			
0–14	130 (12.9)	28 (2.6)	< 0.0001
15–29	321 (32.1)	418 (38.4)	0.002
30–45	317 (31.6)	323 (29.7)	0.33
49–59	136 (13.5)	215 (19.7)	0.002
> 60	99 (9.9)	105 (9.6)	0.86
Nationality			< 0.0001
Saudi	835 (83.3)	449 (41.2)	
Non-Saudi	168 (16.7)	640 (58.8)	
European	5 (2.9)	3 (0.5)	
American	2 (1.2)	1 (0.1)	
African	49 (29.1)	228 (35.6)	
South East Asian	69 (41.2)	213 (33.3)	
Indian Subcontinent	43 (25.6)	195 (30.5)	
Gender			0.03
Male	590 (58.8)	691 (63.4)	
Female	413 (41.2)	398 (36.5)	
AFB Smear			< 0.0001
Positive	367 (36.6)	879 (80.7)	
Negative	636 (63.4)	210 (19.3)	
HIV			0.62
Positive	4 (0.39)	3 (0.27)	
Negative	632 (63.0)	589 (54.1)	
Not Available	371 (37.0)	500 (45.9)	

### Site of extrapulmonary TB infections

**Fig. 1:**
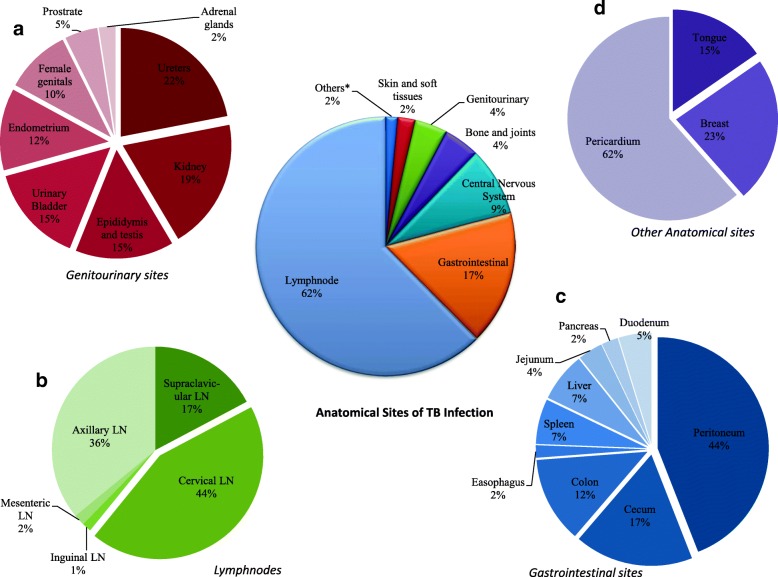
Diversity of extrapulmonary anatomical sites infected with MTBC detected in the study cohort. shows the different extra-thoracic organs or sites infected by MTBC strains. **a**-**d** shows specific sites of infection under genitourinary, lymphnode, gastrointestinal and rare anatomical sites respectively

The reported 1003 EPTB cases were defined into six major groups with a predominance of lymph nodes (62.4%). Other major sites were, gastrointestinal (16.7%), central nervous system (8.6%), genitourinary system (4.1%), skin and soft tissues (2.2%), bone and joints (4.5%) and remaining 1.5% were other rare sites. The cervical (43.6%), axillary (35.9%) and supraclavicular (17.2%) lymph nodes were generally infected. The diversity of infection sites among the study cohort was depicted in Fig. 1.

### Phylogenetic diversity of MTBC isolates of extrapulmonary sites

**Fig. 2:**
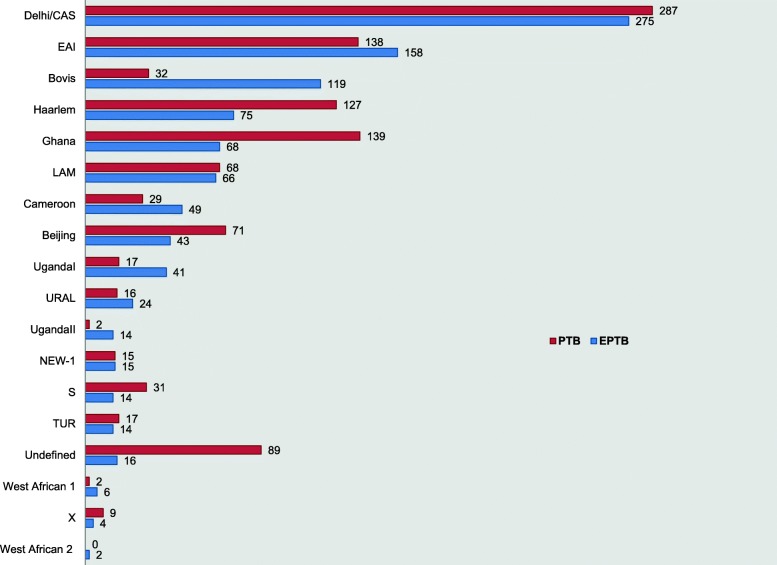
Comparative Lineage variations identified among extra-pulmonary and pulmonary *M.tuberculosis* isolates. The figure shows difference in distribution of lineages defined by combined spoligo and 24 loci MIRU VNTR profiles among pulmonary and extra pulmonary anatomical sites

The MTBC isolates causing EPTB showed a highly diverse lineage spectrum with the presence of all 5 major genetic lineages and 2 sub lineages of lineage 4 (based on large sequence polymorphisms). Interestingly, *M. africanum* lineages West African I (Lineage 5) and West African II (Lineage 6) were also observed. Delhi/CAS (27.4%) (Lineage 3) was predominant followed by EAI (15.7%) (Lineage 1), Haarlem (7.5%) and Ghana (6.8%). In addition, 119 (11.8%) isolates of *M. bovis* and 16 (1.6%) isolates of “undefined” strains have been observed (Fig. 2, Table 2).

**Table 2:** Association of pulmonary and extra pulmonary sites against major strain lineages

Lineages	Total (N/%)	N (%)	N (%)	OR (95% CI)/*P* value
	*N* = 2092	PTB^1^ (*N* = 1089)	EPTB^2^ (*N* = 1003)	
*M. bovis*	*151 (7.3)*	*32 (2.9)*	119 (11.8)	0.22 (0.15–0.33)/ < 0.0001
*Delhi/CAS*	*562 (26.9)*	*287 (26.3)*	275 (27.4)	0.94 (0.78–1.15)/ 0.58
*EAI*	*296 (14.2)*	*138 (12.7)*	158 (15.7)	0.77 (0.60–0.99)/ 0.04
*Beijing*	*114 (5.4)*	*71 (6.5)*	43 (4.3)	1.55 (1.05–2.29)/ 0.02
*Haarlem*	*202 (9.6)*	*127 (11.7)*	75 (7.5)	1.63 (1.21–2.20)/ 0.001
*LAM*	*134 (6.4)*	*68 (6.2)*	66 (6.6)	0.94 (0.66–1.34)/ 0.75
*Cameroon*	*78 (3.7)*	*29 (2.7)*	49 (4.9)	0.53 (0.33–0.85)/ 0.008
*Ghana*	*207 (9.9)*	*139 (12.8)*	68 (6.8)	2.01 (1.48–2.71)/ < 0.0001
*Uganda I*	*58 (2.8)*	*17 (1.6)*	41 (4.1)	0.37 (0.21–0.66)/ 0.0007
*URAL*	*40 (1.9)*	*16 (1.5)*	24 (2.4)	0.60 (0.32–1.1)/ 0.127
*TUR*	*31 (1.5)*	*17 (1.6)*	14 (1.4)	1.12 (0.55–2.28)/ 0.75
*S*	*45 (2.1)*	*31 (2.8)*	14 (1.4)	2.07 (1.09–3.91)/ 0.025
*Uganda II*	*16 (0.76)*	*2 (0.2)*	14 (1.4)	0.13 (0.03–0.57)/ 0.007
*New-I*	*30 (1.4)*	*15 (1.4)*	15 (1.5)	0.92 (0.44–1.89)/ 0.82
*Others* ^*3*^	*144 (6.9)*	*100 (9.2)*	28 (2.8)	–

^1^Pulmonary tuberculosis

^2^Extrapulmonary tuberculosis

^3^Includes lineages, West African I and II, X, New-I and undefined lineages

### Phylogenetic diversity of MTBC isolates in pulmonary tuberculosis

Pulmonary isolates were also phylogenetically diverse with the presence of six defined genetic lineages of MTBC. West African II lineage was absent among the pulmonary cases. The major identified lineage was Delhi/CAS (26.3%) followed by Ghana (12.8%), EAI (12.7%), and Haarlem (11.7%) respectively. Interestingly, 89 (8.2%) isolates could not be defined into any strain lineage based on the combined analysis of spoligo and MIRU typing profiles (Fig. 2, Table 2).

### Comparison of pulmonary and extrapulmonary TB lineage diversity

Comparative analysis of pulmonary and EPTB genotypic data showed similar representation of lineages. However, some of the lineages were over represented among certain sites of infections. Lineages- Ghana, Beijing, Haarlem and S showed a higher affinity towards the pulmonary site of infection. On the other hand, Uganda-I, EAI and Cameroon showed more cases of EPTB. The major lineage among the studied groups Delhi/CAS showed equal rate of presentation in both pulmonary and EPTB cases. *M.bovis* (78.8%) was mostly found among EPTB isolates. Although, total number of cases were less, *M. africanum* strains, West African I and II were found more among extrapulmonary cases. ‘Ancestral’ strains were more common among EPTB (402, 40.1%) compared to PTB (321, 29.5%). Furthermore, ‘Modern’ strains were comparatively higher among PTB cases (679, 62.3%) than EPTB (585, 58.3%) (Fig. 2, Table 2).

### Lineages and associating EPTB sites

We analyzed in detail the adaptability of each major lineage towards different sites of infection. Five major sites such as lymph nodes, gastrointestinal, genitourinary, central nervous systems and bone and joints were analyzed against nine major lineages. Of the 8 major *M.tuberculosis* lineages analyzed against sites of infection Delhi/CAS, EAI, Beijing, Haarlem, LAM and Ghana showed the capability to cause infection to all the sites. On the other hand, Uganda-I and Cameroon were not observed among the rare sites of infection. *M.bovis* infection was highly confined to lymph nodes (88.2%), although other sites also involved (Additional file-).

**Fig. 3:**
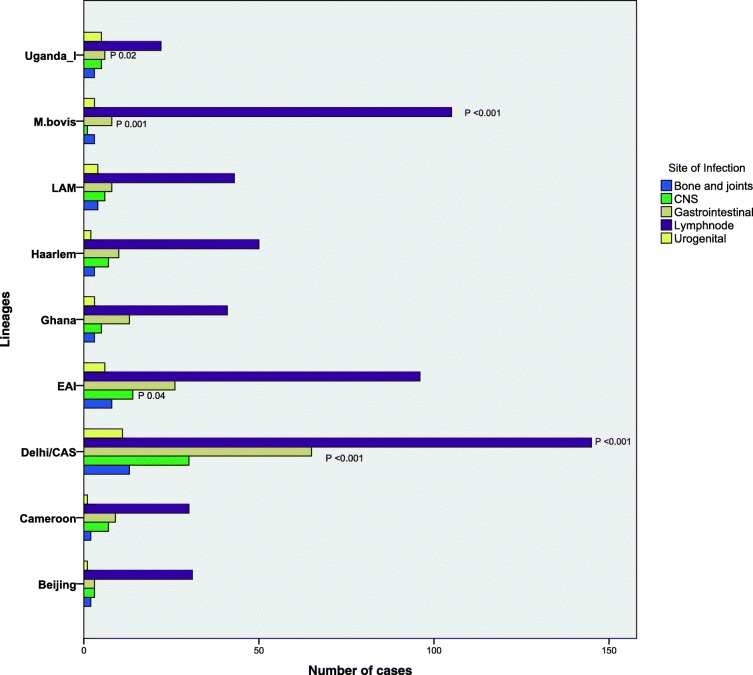
Statistical variability in the prevalence of sites within each lineage. The clustered bar-graph was prepared after chi-square testing with Bonferroni correction for multiplicity to detect the significance of differences in such variations

Statistical analysis based on logistic regression was conducted among selected lineages and extrapulmonary sites of infections. A clustered bar-graph was used to depict the variability in the prevalence of sites within each lineage. The chi-square test was used to detect the significance of the difference in such variations (Fig. 3). The results showed the preponderance of certain lineages to major infection sites such as lymph nodes, gastrointestinal and central nervous systems. Delhi/CAS (Indo-oceanic) was noticed with statistical significance to cause infection in lymph nodes (*P*-value < 0.001; OR 0.57, 95%CI 0.411–0.734) and organs in gastrointestinal systems (*P*-value 0.001; OR 1.87, 95%CI 1.22–2.53). Lineage EAI (East African Indian) also showed a significant association with central nervous system infection sites (*P*-value 0.04; OR 1.98, 95%CI 0.76–3.19). East Asian and Euro American lineages showed no association with any of the analyzed infection sites. However, further analysis on Euro American lineages showed, Uganda-I with an association to gastrointestinal sites (*P*-value 0.02; OR 2.41, 95% CI 0.77–4.06). *M.bovis* also showed statistical significance with, lymph nodes (*P*-value < 0.001; OR 5.22, 95% CI 2.23–8.22) and gastrointestinal sites (*P*-value 0.001; OR 0.33, 95% CI 0.085–0.567). *M. africanum* lineages West African-I and II were few in numbers, thus a detailed analysis was restricted (Fig. 3; Additional file 3).

## Discussion

Relationships between MTBC phylogenetic lineages and clinical site of TB have been analyzed systematically in a cohort of Saudi Arabian patients for the first time. Ahead of the previous studies which reported a primary data on the site of infection and mycobacterial lineages in the country, the current study congregates with a detailed outcome from a large population of diverse patient cohort of 2092 cases [8]. However, previous international studies also showed inconsistent findings on the association of certain phylogenetic lineages with particular infection sites and disease presentation of tuberculosis [6, 7, 12, 13]. The current findings showed positive association of MTBC lineages (Delhi/CAS, EAI, Uganda-I and *M.bovis)* with site of infections such as lymph nodes, gastrointestinal and central nervous systems respectively.

Demographical findings of the cohort showed a domination of Saudi nationals. This finding is inversely proportional to the national tuberculosis data where Non-Saudis were reported with more TB incidences [2]. However, the finding is non-conclusive and the expected reason for this variation is that, opted study centers were four referral hospitals including a military facility which provide services largely to citizens only. The gender of study subjects showed predominance of male, which corroborates with the findings of many recent studies which showed similar trend [2, 8, 14]. In addition, localization of TB manifestations strongly depends on several clinical features. Previously younger age is one of the most common associated with extrapulmonary infections [15–17]. The current findings also corroborate with previous studies and showed statistically significant predominance of younger age towards EPTB.

HIV positivity in the cohort was very much limited (0.33%), although 58.5% of the total cases only underwent the testing. This finding is in concordance with the low rate of annually reported HIV positive cases among the local population (1.5 cases per 100,000 for Saudis) in the country [18]. HIV testing data was inconsistently recorded (46% not tested) in the study, while untreated HIV infection is well recognized as an important factor in determining an extrapulmonary clinical phenotype, therefore its influence on clinical phenotype could not be analyzed [17].

The site of infections followed very detailed classification and reported as highly diverse with several rare sites. Lymph node was the most commonly infected sites followed by the gastrointestinal, osteoarticular and central nervous systems. This finding shows similar trend reported in a recent Saudi Arabian and international studies [3, 14, 19]. The current cohort of EPTB cases revealed several rare infection sites such as bone marrow, breast, tongue, testis, kidney, prostate glands, spleen, pancreas, duodenum, cecum and jejunum as observed in available literature [20, 21].

The major advantage of our data compared to previously published studies with limited sample size and sample diversity was the presence of all defined lineages. Phylogenetic diversity of pulmonary and extrapulmonary isolates showed the presence of all defined MTBC lineages (Lineage 1–7) in the country. Interestingly, for the first time in the country, presence of *M. africanum* lineages West African I and II were noticed*. M.africanum* has not been detected in any of the previous nationwide studies [8–10]. Saudi Arabia annually receives approximately 8–10 millions of pilgrims (from 184 countries) from TB endemic regions, in addition to its migrant workers population of 10.4 million from around the world. This massive influx of foreign nationals solely induces a higher impact on the population structure of MTBC in the country [10, 22]. The increased possibilities of TB transmission and exportation in mass gathering particularly during Hajj were highly projected [23]. Therefore, the higher diversity of strain lineages in the study could be well explained.

Comparative analysis on the strain diversity between PTB and EPTB was a key objective of the study. The findings showed the presence of all the lineages among both groups though the proportion varied. Ancestral lineages were found relatively high among EPTB cases, which are in concordance with previously published studies from other part of the world including TB endemic regions [24, 25]. On the other hand, “Modern” strains were more common among PTB. Predominance of “Modern” strains among the pulmonary cases in the country was previously well documented, corroborating with other global regions [6, 8, 13].

Detailed statistical analysis to find an organ or site specific preponderance of MTBC lineages showed significant association of ancestral lineages, Delhi/CAS and *M.bovis* to lymphadenopathy and gastrointestinal TB. Although, previous studies showed predominant isolation of Delhi/CAS and *M.bovis* from lymph nodes and gastrointestinal sites only few of them were statistically significant [24, 26, 27]. Interestingly, association of lineage EAI with central nervous system TB which mainly included tuberculous meningitis (TBM) was significant. Although previous studies showed the mycobacterial genotypes could play a major role in disease severity, emergence of drug resistance, host response and transmissibility in TBM cases, findings of statistical significance between certain lineages and CNS disease sites are highly scarce [28, 29]. Association of Uganda-I lineage to gastro intestinal sites of infection was another significant finding. The Uganda-I lineage has not been studied in detail as a cause of EPTB and therefore very much limited information is only available in literature on its association with any clinical manifestation [30].

The influence of genomic diversity on pathobiological properties such as transmissibility, virulence, immune responses, and clinical manifestations were established in several previous studies [6, 31, 32]. In addition, lineage specific difference in mycobacterial transcriptomic responses, cytokine induction patterns in animal or cellular infection models also were proved in recent studies [33–35]. One of the most intensively studied lineage Beijing, showed several potential mechanisms as evidences of associations towards clinical manifestations [36]. In animal models less protective Th1 response and high virulence has been documented when infected with Beijing strains [35]. Other proposed mechanisms in Beijing strains, which increase the pathobiological adaptations and phenotypic stability are *DosR* up- regulation and production of a phenolic glycolipid by an intact *pks15–1* [37, 38]. However, the real mechanisms behind all such associations particularly on less frequently encountered lineages are still not well established.

The study has few limitations mostly related to sampling and data analysis. Although, the study population largely consisted of Saudi nationals it cannot be considered as a nationwide population based analysis. The cohort was a selective group of citizens and mostly from the central region of the country. The expatriate population was not well represented mainly due to their eligibility of treatment in study centers although they represent 33% of total population of the country. Analysis of confounding factors behind the EPTB in the current cohorts was limited and this was not included as a major objective of the study. In addition, 5.1% of the total isolates in the study could not be assigned into “defined” lineage even after combining the MIRU and spoligotyping techniques, and no alternative technique was utilized to define their phylogenetic nature.

## Conclusions

In conclusion, as the first systematic large study of its type from the Middle East, the findings showed the extreme genotypic diversity of MTBC in extrapulmonary sites of infections. Statistically significant association of infection sites with lineages such as Delhi/CAS, EAI, Uganda-I and *M.bovis* could be established. Moreover, these findings support the view that MTBC strains within individual genotypic lineages might have evolved unique pathogenic characteristics that are capable of influencing the clinical outcome of the infection.

## Additional files

Additional file 1: Nationality stratified by site of infection. Table shows the distribution of pulmonary and extrapulmonary cases among 39 nationalities included in the study. (PDF 194 kb)

Additional file 2: Nationality of the patient and distribution of lineages. The figure shows the distribution of major MTBC lineages among top 11 nationalities. (PDF 277 kb)

Additional file 3: Statistical association of major MTBC lineages and extrapulmonary site of infections. (PDF 112 kb)

## Abbreviations

CAS: Central Asian
EAI: East African Indian
EPTB: Extrapulmonary tuberculosis
MIRU: Mycobacterial Interspersed Repetitive Units
MTBC: Mycobacterium tuberculosis complex
PTB: Pulmonary tuberculosis
TB: Tuberculosis
TBM: Tuberculous meningitis
