# Supplementary material for: Impact of Mycobacterium tuberculosis complex lineages as a determinant of disease phenotypes from an immigrant rich moderate tuberculosis burden country
Source: Respir Res. 2018 Dec 27;19:259. doi: 10.1186/s12931-018-0966-x (PMC6307224; doi:10.1186/s12931-018-0966-x)
Supplement: Supplementary file 1 — Nationality stratified by site of infection. Table shows the distribution of pulmonary and extrapulmonary cases among 39 nationalities included in the study. (PDF 194 kb) [file 12931_2018_966_MOESM1_ESM.pdf]

| | Nationality | EPTB | PTB | Total | Goegraphical Origin |
|---|---|---|---|---|---|
| 1 | Saudi | 835 | 449 | 1284 | **Saudi** |
| 2 | France | 1 | 2 | 3 | |
| 3 | Poland | 1 | 1 | 2 | |
| 4 | Romania | 1 | 1 | 2 | **Europe** |
| 5 | Czeck | 2 | 1 | 3 | |
| 6 | USA | 2 | 1 | 3 | **America** |
| 7 | Ethiopia | 8 | 31 | 39 | |
| 8 | Somalia | 6 | 27 | 33 | |
| 9 | Chad | 2 | 25 | 27 | |
| 10 | Sudan | 3 | 23 | 26 | **Africa** |
| 11 | Nigeria | 3 | 20 | 23 | |
| 12 | Erytrea | 3 | 16 | 19 | |
| 13 | Algeria | 2 | 8 | 10 | |
| 14 | Libya | 1 | 6 | 7 | |
| 15 | Senegal | 3 | 5 | 8 | |
| 16 | Zambia | 1 | 3 | 4 | |
| 17 | Morocco | 3 | 6 | 9 | |
| 18 | Madagascar | 1 | 7 | 8 | |
| 19 | SierraLeone | 3 | 11 | 14 | |
| 20 | Cameroon | 2 | 9 | 11 | |
| 21 | Ghana | 2 | 8 | 10 | |
| 22 | Djibouti | 2 | 5 | 7 | |
| 23 | South Africa | 1 | 3 | 4 | |
| 24 | Kenya | 3 | 6 | 9 | |
| 25 | Egypt | 5 | 12 | 17 | |
| 26 | Syria | 3 | 8 | 11 | |
| 27 | Lebanon | 1 | 3 | 4 | **Middle East** |
| 28 | Yemen | 11 | 28 | 39 | |
| 29 | Indonesia | 26 | 90 | 116 | |
| 30 | Philippines | 19 | 78 | 97 | |
| 31 | Malayasia | 3 | 7 | 10 | **South East Asia** |
| 32 | Vietnam | 1 | 4 | 5 | |
| 33 | India | 11 | 61 | 72 | |
| 34 | Pakistan | 18 | 56 | 74 | |
| 35 | Bangladesh | 9 | 24 | 33 | |
| 36 | Srilanka | 2 | 17 | 19 | |
| 37 | Nepal | 1 | 10 | 11 | **Indian Sub-continent** |
| 38 | Myanmar | 1 | 8 | 9 | |
| 39 | Afghanistan | 1 | 9 | 10 | |
| | | 1003 | 1089 | 2092 | |
