# Supplementary figures and images for: Impact of Mycobacterium tuberculosis complex lineages as a determinant of disease phenotypes from an immigrant rich moderate tuberculosis burden country
Source: Respir Res. 2018 Dec 27;19:259. doi: 10.1186/s12931-018-0966-x (PMC6307224; doi:10.1186/s12931-018-0966-x)

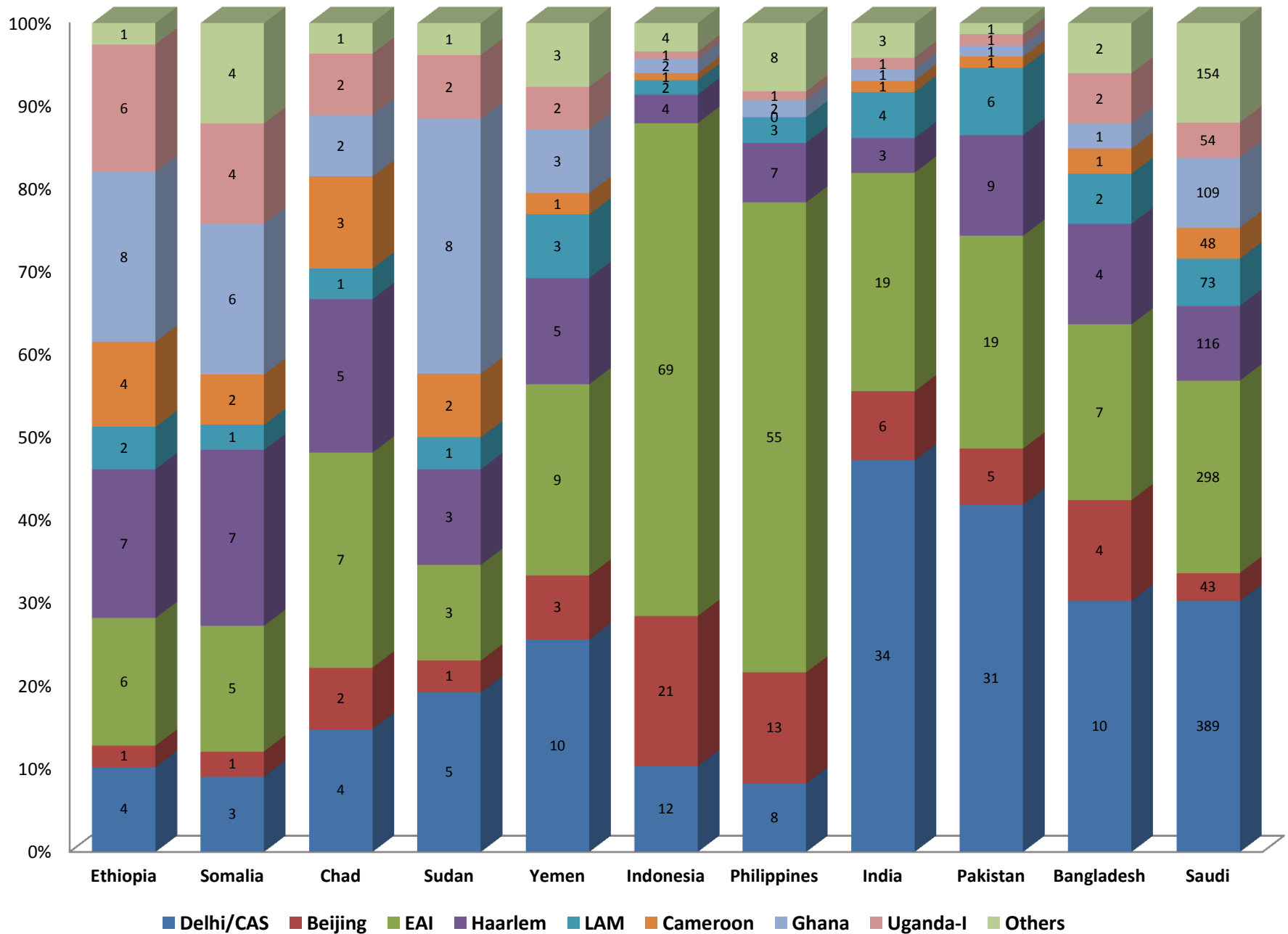

Supplement: Supplementary file 2 — Nationality of the patient and distribution of lineages. The figure shows the distribution of major MTBC lineages among top 11 nationalities. (PDF 277 kb) [file 12931_2018_966_MOESM2_ESM.pdf]
