# Supplementary material for: Impact of Mycobacterium tuberculosis complex lineages as a determinant of disease phenotypes from an immigrant rich moderate tuberculosis burden country
Source: Respir Res. 2018 Dec 27;19:259. doi: 10.1186/s12931-018-0966-x (PMC6307224; doi:10.1186/s12931-018-0966-x)
Supplement: Supplementary file 3 — Statistical association of major MTBC lineages and extrapulmonary site of infections. (PDF 112 kb) [file 12931_2018_966_MOESM3_ESM.pdf]

Supplimentary Table-2: Correlation of major MTBC lineages and site of extrapulmonary infections.

Lineages	*N (%)* EPTB[1] *(N=1003)*	*Lymphnode N=626*	*OR(95% CI):p value*	GITB[2] *N=168*	*OR(95% CI):p value*	CNSTB[3] *N=86*	*OR(95% CI):p value*	*Bone & joints N=45*	*OR(95% CI):p value*	UGTB[4] *N=41*	*OR(95% CI):p value*	*Others N=37*
M.bovis	119(11.8)	105(16.8)	**5.22(2.23-8.22):<0.001**	8(4.8)	**0.33(0.085-0.567): 0.001**	-		3(6.7)	0.52(0.16-1.69): 0.38	3(7.3)	0.57(0.17-1.89): 0.22	-
Delhi/CAS (Indo Oceanic)	275(27.4)	145(23.2)	**0.57(0.411-0.734):<0.001**	65(38.7)	**1.87(1.22-2.53): <0.001**	30(34.9)	0.74(0.42-1.06): 0.18	13(28.9)	1.07(0.37-1.79): 0.82	11(26.8)	11.39(2.49-20.28)	11(29.7)
EAI (East African Indian)	158(15.7)	96(15.3)	0.92(0.59-1.24): 0.62	26(15.5)	0.97(0.52-1.42): 0.91	14(16.3)	**1.98(0.76-3.19):0.04**	8(17.8)	1.16(0.79-1.53): 0.86	6(14.6)	1.66(1.29-2.02): 0.39	8(21.6)
Beijing (East Asian)	43(4.3)	31(4.9)	1.58(0.52-2.64): 0.24	3(1.8)	0.36(0.11-1.18): 0.08	3(3.5)	0.79(0.24-2.61)	2(4.4)	1.04(0.24-4.44)	1(2.4)	0.58(0.07-4.29)	3(8.1)
Euro American[5]	400(39.8)	245(39.1)	0.92(0.79-1.19):0.54	66(39.3)	0.97(0.69-1.36):0.86	37(43)	1.15(0.74-1.80):0.53	18(40)	1.00(0.55-1.85):0.88	20(48.8)	1.48(0.78-2.72):0.24	15(40.5)
-Haarlem	75(7.5)	50(7.9)	1.22(0.61-1.83): 0.50	10(5.9)	0.75(0.23-1.26): 0.51	7(8.1)	1.10(0.20-200): 0.98	3(6.7)	0.88(0.26-2.90)	2(4.9)	0.62(0.15-2.63)	3(8.1)
-LAM	66(6.6)	43(6.9)	1.13(0.54-1.72): 0.73	8(4.8)	0.67(0.16-1.18): 0.38	6(6.9)	1.07(0.44-2.07): 0.88	4(8.9)	1.41(0.48-4.06): 0.74	4(9.7)	1.56(0.54-4.54)	1(2.7)
-Cameroon	49(4.9)	30(4.8)	0.95(0.39-1.51): 0.98	9(5.3)	1.12(0.29-1.96): 0.90	7(8.1)	1.84(0.31-3.38): 0.23	2(4.4)	0.90(0.21-3.83)	1(2.4)	0.47(0.06-3.53): 0.71	-
-Ghana	68(6.8)	41(6.5)	1.94(0.97-2.92): 0.80	13(7.7)	1.18(0.44-1.93): 0.71	5(5.8)	0.84(0.05-1.62): 0.88	3(6.7)	0.98(0.29-3.25)	3(7.3)	1.09(0.32-3.62)	3(8.1)
-Uganda I	41(4.1)	22(3.5)	0.68(0.25-1.12): 0.31	6(3.6)	**2.41(0.77-4.06): 0.02**	5(5.8)	1.51(0.06-2.96): 0.57	3(6.7)	1.73(0.51-5.83): 0.61	5(12.2)	3.57(0.02-7.12): 0.06	-
Others[6]	109(10.87)	53(7.34)	-	20(11.9)	-	9(10.5)	-	7(15.5)	-	5(12.2)	-	8(21.6)

[1] Extrapulmonary tuberculosis

[2] Gastrointestinal tuberculosis

[3] Central nervous system tuberculosis

[4] Urogenital tuberculosis

[5] Lineages other than Delhi/CAS, EAI, Beijing, West African I and II and Bovis together grouped. Sub grouped to analyze 5 major clades

[6] Lineages including Uganda II, West African I and II, X, New-I, S, TUR and undefined lineages
